# Expression Characteristics of PAX7 and Its Prognostic Correlation in Breast Cancer

**DOI:** 10.1155/tbj/3060151

**Published:** 2026-01-11

**Authors:** Bahatiguli Silafu, Jinxing Huang, Xierzhati Aizezi

**Affiliations:** ^1^ Department of Physician Services, Xinjiang International Medical Center (Xinjiang International Hospital), Urumqi, Xinjiang, China; ^2^ Department of Urology, The Fifth Affiliated Hospital of Xinjiang Medical University, Urumqi, Xinjiang, China, xjmu.edu.cn

**Keywords:** biomarker, breast cancer, PAX7 gene, prognosis

## Abstract

**Objective:**

To conduct a thorough analysis of public databases to investigate the expression patterns of the PAX7 gene in breast cancer.

**Methods:**

We gathered gene expression data, clinical details, immunohistochemistry images, and genomic information from breast cancer patients through various public databases, such as TCGA, THPA, GEPIA, and cBioPortal. To analyze differential expression, we used the limma package. We assessed the relationship between PAX7 and clinical characteristics using chi‐square tests and Fisher’s exact tests. For survival analysis, we employed Kaplan–Meier survival curves and Cox regression models to evaluate prognostic factors. Furthermore, we conducted functional clustering analysis to explore the roles of related genes. The MCPcounter and TIMER packages were utilized for analyzing immune infiltration, while statistical computations and visualizations were carried out using the R programming language and its associated packages.

**Results:**

The expression levels of PAX7 in breast cancer tissues were significantly higher than normal tissues. Survival analyses showed that patients with high PAX7 expression had notably lower overall survival, disease‐specific survival, and progression‐free survival, establishing PAX7 as an independent prognostic factor. Assessments of immune infiltration demonstrated a significant relationship between PAX7 and the levels of Th2 cells, TReg, and TFH. Additionally, Clustering analysis of PAX7 genes showed enrichment in cell division, chromosome regions, and pathways like the cell cycle.

**Conclusion:**

PAX7 was highly expressed in cancer tissues and had a notable impact on patient survival. Furthermore, it was identified as an independent prognostic factor, with related genes being enriched in various biological processes.

## 1. Introduction

Breast cancer, known for its high incidence and continuous rise, especially among women, has become a major public health issue worldwide [1–3]. This increase is not only seen in developed countries but is also becoming more evident in developing nations, raising serious concerns about breast cancer prevention and treatment [4, 5]. Surgical treatments, which are commonly used, aim to remove tumor tissues but often lead to changes in body shape, creating psychological challenges for patients [6]. Although therapies such as radiotherapy and chemotherapy target cancerous cells, they do a great deal of harm to healthy tissues, such as hair loss and digestive problems, greatly lowering the quality of life a patient has [7]. More alarmingly, even after comprehensive treatments, recurrence and metastasis are still threatening breast cancer patients and are critical factors that limit long‐term survival and improvement in prognosis [8, 9].

A great degree of heterogeneity underlies the very process of emergence and development regarding breast cancer, which is characterized by aberrant regulation of various genes with complex signaling pathways [10]. This inherent variability in the manifestation of a disease complicates current treatment strategies and leads to great variability in therapeutic outcomes among patients [11]. Recent studies highlight the functional heterogeneity of PAX7 across cancer types: in solid tumors like rhabdomyosarcoma and glioblastoma, PAX7 overexpression drives oncogenic transformation and metastasis, whereas in hematological malignancies such as acute myeloid leukemia, its role remains context‐dependent, potentially suppressing differentiation or promoting survival depending on genetic codrivers [12, 13]. The need exists to perform comprehensive investigations scientifically and clinically concerning the newest biomarkers and therapeutic targets to provide early and differential diagnosis, personalized treatment planning, and reliable prognosis assessment. One promising area of research is the PAX7 gene, which has shown a vital role in the progression of several cancer types [14, 15]. Emerging mechanistic insights reveal that PAX7 activity is tightly regulated by posttranslational modifications, including acetylation and ubiquitination, which modulate its stability and DNA‐binding capacity. For instance, MYST1‐mediated acetylation of PAX7 at lysine residues enhances its transcriptional activity, while SIRT2 deacetylation promotes proteasomal degradation, creating a dynamic regulatory loop [13]. Additionally, epigenetic alterations, such as DNA methylation at PAX7 enhancer regions, have been linked to its dysregulation in therapy‐resistant tumors [16]. Although PAX7 has been implicated in a wide range of cancers, its biological function and molecular mechanisms in breast cancer have not yet been fully understood. PAX7, as a transcription factor, regulates the expression of a wide array of downstream target genes modulating key biological processes (BPs) such as cell proliferation, differentiation, and apoptosis, all of which contribute to the course of tumorigenesis [17, 18]. Notably, PAX7 has recently been implicated in maintaining cancer stemness by activating Wnt/β‐catenin and Notch pathways, thereby enriching chemotherapy‐resistant subpopulations in triple‐negative breast cancer. Its interaction with the tumor microenvironment, particularly through TGF‐β‐SMAD‐mediated epithelial–mesenchymal transition, further underscores its role in metastatic progression [19, 20]. Therefore, a systematic investigation into expression patterns, biological functions, and relation to clinical features and prognosis would make available new information on PAX7 in the pathogenesis of breast cancer and support novel therapeutic strategies.

This study will try to delve into how the PAX7 gene is expressed in breast cancer and its implications for the prognosis of the patients by analyzing various public databases. The strength of this approach is that it can collate a number of datasets, hence adding strength and applicability to the findings. With advanced statistical analysis, such as survival and Cox regression models, it will be explained how the PAX7 signature correlated with several clinical characteristics and overall survival (OS) in breast cancer patients. The main aim is the establishment of the potential of PAX7 as a biomarker that will be highly useful for early diagnosis and targeted treatment with better clinical management and therapeutic strategy in breast cancer.

## 2. Materials and Methods

### 2.1. Data Sources and Preprocessing

Data for this study were obtained from publicly available databases to ensure comprehensiveness and reliability. Gene expression data and clinical information on breast cancer patients were provided by the Cancer Genome Atlas (TCGA) at https://www.cancer.gov/tcga, whereas the Human Protein Atlas (THPA) was utilized to download immunohistochemistry (IHC) images representing PAX7 protein in normal and in breast cancer tissues at https://www.proteinatlas.org/. We employed the GEPIA database (http://gepia.cancer-pku.cn/) to analyze the association between PAX7 expression and overall survival (OS) in breast cancer patient. Genomic data on the distribution of genomic alteration in PAX7 within cases of breast cancer were derived from cBioPortal, https://www.cbioportal.org/. After quality control, data preclusion, including removing the low‐quality samples, was allowed to undergo normalization of the expression data. For pan‐cancer analyses, we extended data acquisition to include 33 cancer types from TCGA.

To ensure representativeness of TCGA breast cancer cohorts, cases and adjacent normal controls were matched by age, self‐reported ethnicity, and molecular subtypes (luminal A/B, HER2‐enriched, and triple‐negative) using the TCGA Clinical Data Resource. To address potential batch effects from multidatabase integration, we implemented a two‐stage harmonization protocol: (1) intra‐dataset normalization using sva for THPA image quantification scores and (2) cross‐dataset adjustment via ComBat‐seq (from the sva package) for RNA‐seq expression matrices prior to differential expression analysis.

### 2.2. Differential Expression Analysis

Differential expression was analyzed using the limma package of R to find out the difference in the expression of PAX7 between breast cancer tissues and normal tissues. Here, we selected the significant genes with criteria |log2FoldChange| > 2 and adjusted *p* value less than 0.05 to ensure that the selected genes were biologically relevant. For cross‐cancer comparisons, expression data were standardized using ComBat‐seq to remove batch effects, followed by log2 (TPM + 1) transformation. Multicancer differential expression analysis applied a stringent false discovery rate (FDR) correction of < 0.01 to account for hypothesis testing across 33 cancer types.

### 2.3. Pan‐Cancer Analysis Workflow

A dedicated pan‐cancer analysis workflow was implemented to ensure consistency across cancer types. This included (1) uniform preprocessing of RNA‐seq data using the Toil pipeline (UCSC Xena Platform) to generate harmonized TPM values; (2) batch correction via the sva package for technical variability across TCGA cohorts; and (3) cross‐cancer normalization using quantile normalization at the gene level. The pan‐cancer significance threshold (|log2FoldChange| > 1.5, FDR < 0.05) was determined through simulation‐based power analysis to balance sensitivity and specificity in multicancer comparisons. Additional corrections for tumor purity and stromal contamination were applied using ESTIMATE algorithm–adjusted expression values.

### 2.4. Correlation Analysis With Clinical Characteristics

The correlations of PAX7 expression with age, pathological staging, HER2 status, PR, ER, PAM, and OS status were analyzed by chi‐square tests or Fisher’s exact tests. The correlations were further visualized using the ggplot2 package in R. *p* < 0.05 was considered statistically significant unless otherwise specified.

### 2.5. Survival Analysis

We performed Kaplan–Meier survival plots and log‐rank tests to determine the correlation between PAX7 expression and OS in patients with breast cancer. The median level of PAX7 expression was used to divide patients into high‐ and low‐expression groups for a comparison of survival. Furthermore, PAX7 expression was analyzed for association with disease‐specific survival (DSS) and progression‐free interval (PFI) based on data obtained from the TCGA database.

### 2.6. Cox Regression Model

Both univariate and multivariate Cox proportional hazards models were constructed to assess the independent effect that the PAX7 expression and other clinical features may have on the prognosis of breast cancer patients. *p* < 0.05 in the univariate analysis was used to include variables in the multivariate model. Variable selection into the multivariate model was by the forward stepwise regression method.

### 2.7. Nomogram Construction and Validation

We then utilized the rms package in R to establish a nomogram that integrates all the independent prognostic factors identified by multivariate Cox regression analysis for the prediction of OS rates at 1, 5, and 10 years in breast cancer patients. The nomogram is developed directly from the Cox model regression coefficient point system and the calibration curves to validate these predictions.

### 2.8. Functional Clustering Analysis

The clusterProfiler package in R facilitated the functional clustering analysis of genes coexpressed with PAX7, encompassing BPs, cellular components (CCs), and molecular functions (MFs), along with KEGG pathway enrichment analysis. A statistical significance threshold of *p* < 0.05 was established to ensure the reliability of the analysis outcomes.

### 2.9. Immune Infiltration Analysis

Utilizing the MCPcounter and TIMER packages in R, we evaluated the association between PAX7 expression and the extent of immune cell infiltration within the tumor microenvironment. We compared the infiltration levels of various immune cell types in samples from patients exhibiting high versus low PAX7 expression to investigate the potential relationship between PAX7 and the immune microenvironment.

### 2.10. Statistical Software and Graphical Representation

The statistical analyses conducted in this study primarily utilized the R programming language (Version 4.0.2). Specifically, the limma package was employed for differential expression analysis, the survival package was employed for survival analysis and Cox regression models, the rms package was employed for the construction and calibration curve of the nomogram, and the ggplot2 package was employed for producing high‐quality visual representations. Furthermore, online tools and databases such as GEPIA, THPA, GEPIA, and cBioPortal were instrumental in supporting specific data analysis and visualization tasks. All statistical tests were conducted at a significance threshold of *p* < 0.05, with ^∗^ representing *p* < 0.05, ^∗∗^ denoting *p* < 0.01, and ^∗∗∗^ indicating *p* < 0.001.

## 3. Results

### 3.1. Analysis of PAX7 Expression Patterns

In this investigation, we performed a comprehensive analysis of PAX7 expression patterns in breast cancer, revealing significant disparities compared to normal tissues. As illustrated in Figure 1(a), we noted variations in PAX7 expression across different cancer types, while the heatmap depicted in Figure 1(b) showcased the distribution of differentially expressed genes. The volcano plot displayed in Figure 1(c) highlighted the statistical significance of differential gene expression. Figures 1(d) and 1(e) illustrate the expression differences of PAX7 between breast cancer patients and normal individuals, with PAX7 expression significantly elevated in breast cancer cases (*p* < 0.001). Immunohistochemical staining results (Figures 1(f) and 1(g)) indicated that PAX7 staining intensity was greater in breast cancer tissues compared to normal tissues. Lastly, the cBioPortal OncoPrint presented in Figure 1(h) depicted the distribution of genomic alterations concerning PAX7 in breast cancer patients, which may correlate with the regulation of PAX7 expression.

Figure 1Expression levels of PAX7. (a) The differential expression of PAX7 in diverse cancers. (b) Heatmap of differentially expressed genes. (c) Volcano map of differential genes. (d) Discrepancy in PAX7 expression between breast cancer and normal individuals. (e) Variation in PAX7 expression between breast cancer and normal samples. (f, g) IHC staining of PAX7 in normal tissues and breast cancer. (h) cBioPortal OncoPrint graph depicting the distribution of PAX7 genomic alterations in breast cancer patients. ^∗^ *p* < 0.05, ^∗∗^ *p* < 0.01, and ^∗∗∗^ *p* < 0.001.

**Figure:**
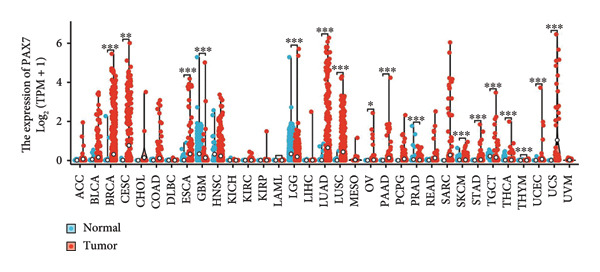
(a)

**Figure:**
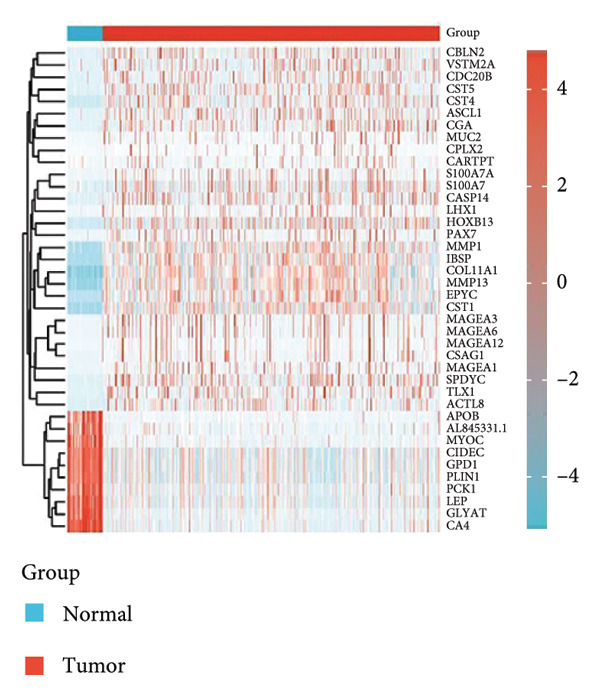
(b)

**Figure:**
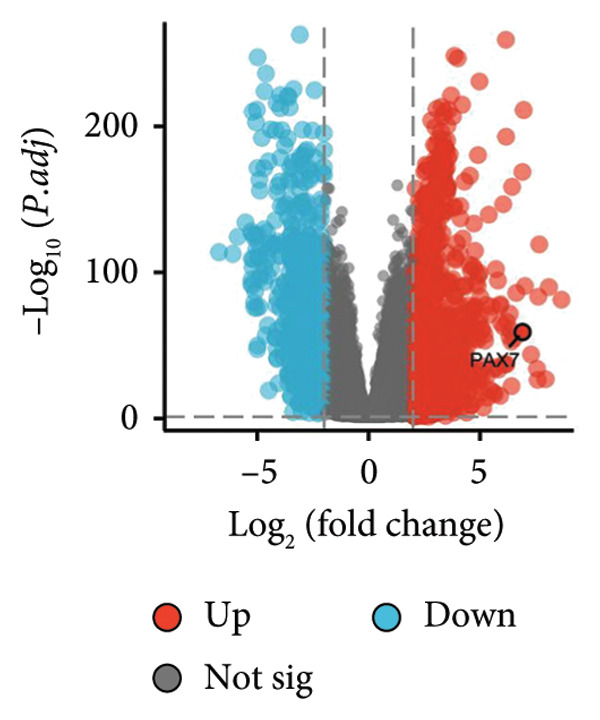
(c)

**Figure:**
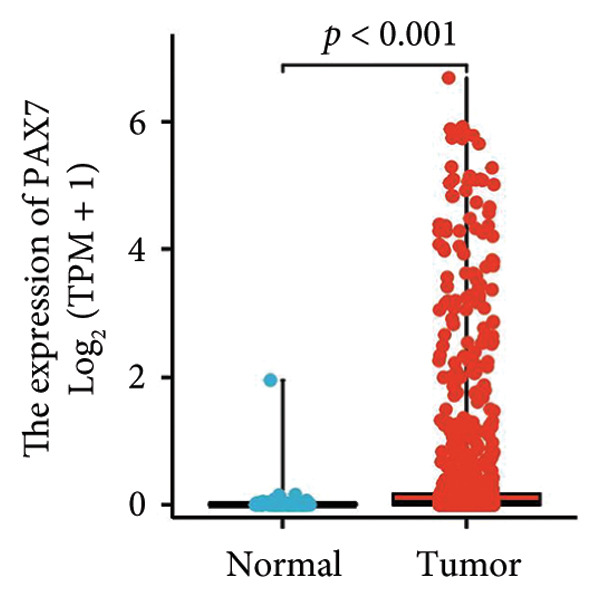
(d)

**Figure:**
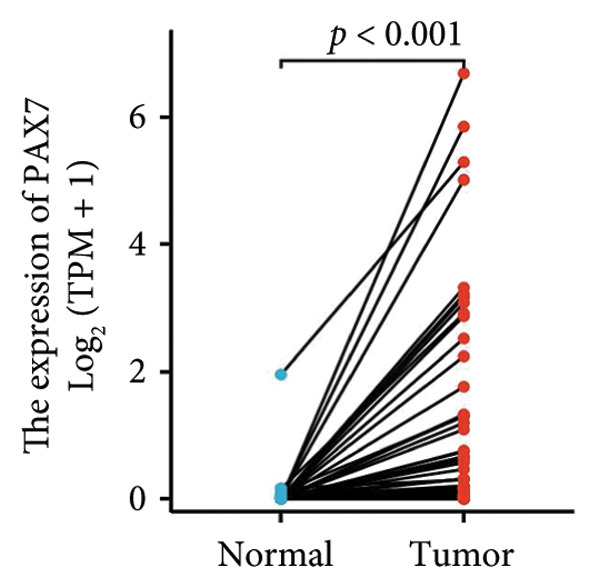
(e)

**Figure:**
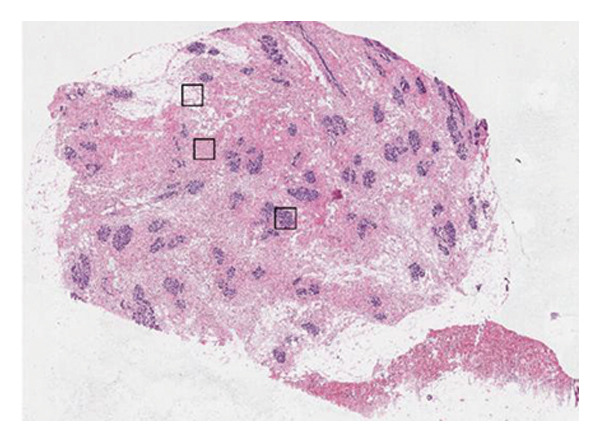
(f)

**Figure:**
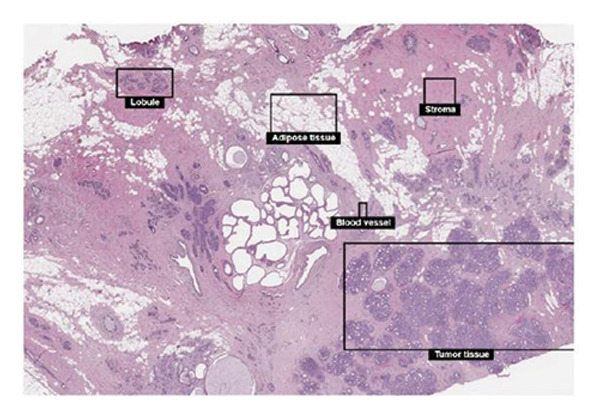
(g)

**Figure:**
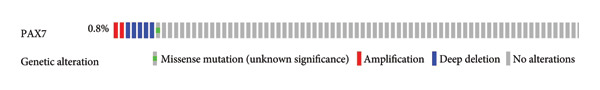
(h)

### 3.2. Correlation Between PAX7 and Clinical Characteristics

We conducted a statistical analysis of the baseline data from 1087 breast cancer patients sourced from TCGA database (Table 1). The data were categorized into two groups: 543 breast cancer patient samples exhibiting relatively low PAX7 expression and 544 samples with comparatively high PAX7 expression. Table 1 indicates that PAX7 expression was closely associated with various clinical characteristics associated with pathological N staging (*p* = 0.031), pathological stage (*p* = 0.031), HER2 positive status (*p* < 0.001), PAM50 subtypes (*p* < 0.001), and poor OS (*p* = 0.009), while showing no significant correlation with pathological T staging (*p* = 0.218) and PR status (*p* = 0.965). Figure 2 illustrates the associations between PAX7 expression and tumor T, N, and M staging, pathological stage, OS events, HER2, PR, ER status, and PAM50 subtypes, demonstrating that PAX7 is significantly upregulated in breast cancer and linked to various clinical characteristics (*p* < 0.05).

**Table 1:** Correlation between PAX7 expression and clinical characteristics of breast cancer.

Characteristics	Low expression of PAX7	High expression of PAX7	*p* value
*n*	543	544	
Age, *n* (%)			0.881
> 60	243 (22.4%)	241 (22.2%)	
≤ 60	300 (27.6%)	303 (27.9%)	
Pathologic T stage, *n* (%)			0.218
T1	153 (14.1%)	125 (11.5%)	
T2	310 (28.6%)	321 (29.6%)	
T3	65 (6%)	75 (6.9%)	
T4	15 (1.4%)	20 (1.8%)	
Pathologic N stage, *n* (%)			0.031
N0	264 (24.7%)	252 (23.6%)	
N1	192 (18%)	167 (15.6%)	
N2	45 (4.2%)	71 (6.6%)	
N3	34 (3.2%)	43 (4%)	
Pathologic M stage, *n* (%)			0.073
M1	6 (0.6%)	14 (1.5%)	
M0	455 (49.2%)	450 (48.6%)	
Pathologic stage, *n* (%)			0.031
Stage IV & Stage III	116 (10.9%)	146 (13.7%)	
Stage II & Stage I	416 (39.1%)	385 (36.2%)	
HER2 status, *n* (%)			< 0.001
Positive	47 (6.4%)	110 (15.1%)	
Negative & indeterminate	320 (43.9%)	252 (34.6%)	
PR status, *n* (%)			0.965
Positive	345 (33.2%)	347 (33.4%)	
Negative & indeterminate	172 (16.6%)	174 (16.8%)	
ER status, *n* (%)			0.070
Positive	385 (37.1%)	412 (39.7%)	
Negative & indeterminate	133 (12.8%)	109 (10.5%)	
PAM50, *n* (%)			< 0.001
Normal	19 (1.7%)	21 (1.9%)	
LumA	305 (28.1%)	259 (23.8%)	
LumB	73 (6.7%)	133 (12.2%)	
Her2	13 (1.2%)	69 (6.3%)	
Basal	133 (12.2%)	62 (5.7%)	
OS event, *n* (%)			0.009
Dead	61 (5.6%)	91 (8.4%)	
Alive	482 (44.3%)	453 (41.7%)	

Figure 2PAX7 expression in TCGA tumor samples by clinical features. (a) T stage, (b) N stage, (c) M stage, (d) pathological stage, (e) OS events, (f) HER2 status, (g) PR status, (h) ER status, and (i) PAM50. ^∗^ *p* < 0.05, ^∗∗^ *p* < 0.01, and ^∗∗∗^ *p* < 0.001.

**Figure:**
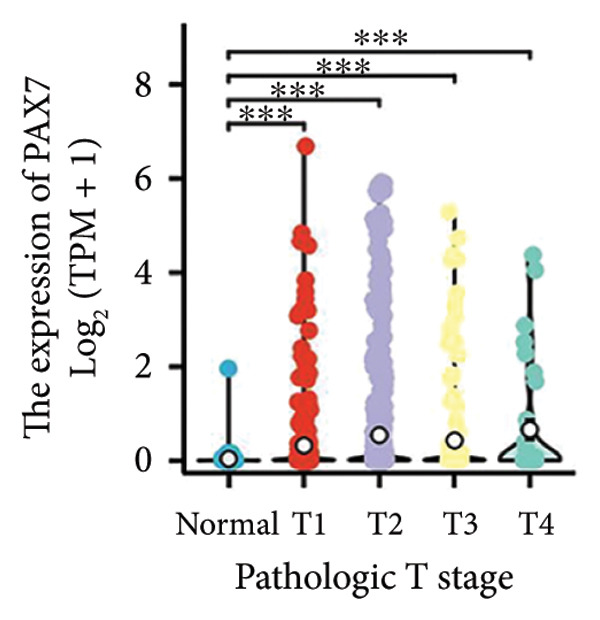
(a)

**Figure:**
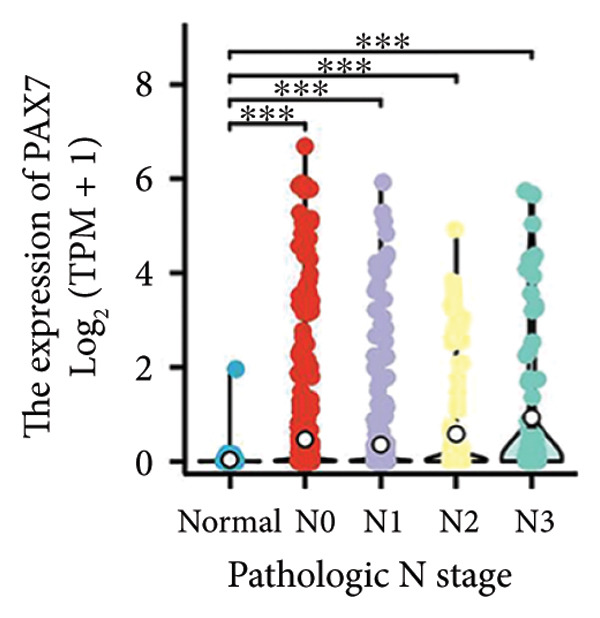
(b)

**Figure:**
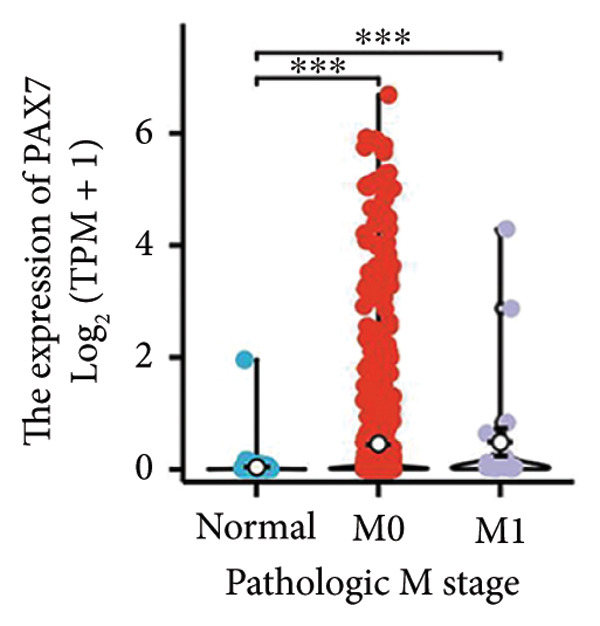
(c)

**Figure:**
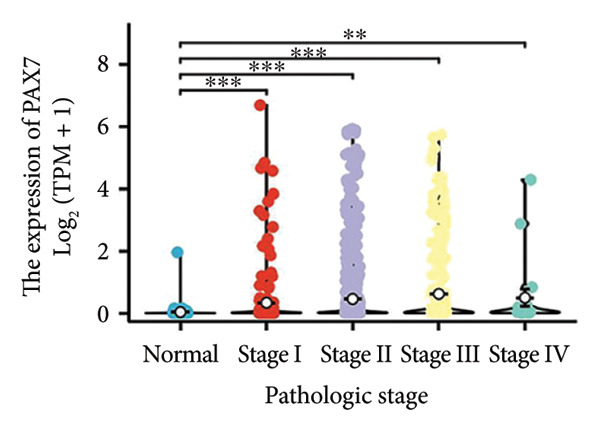
(d)

**Figure:**
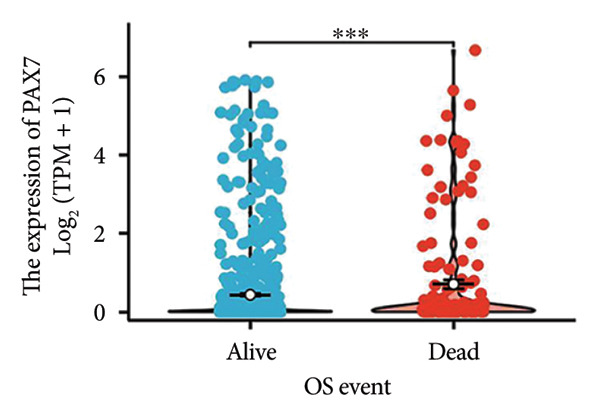
(e)

**Figure:**
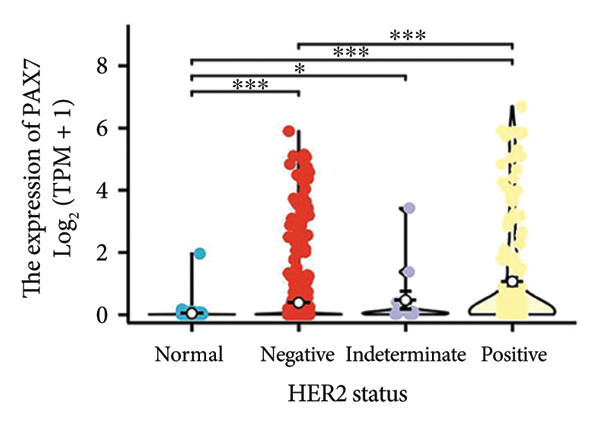
(f)

**Figure:**
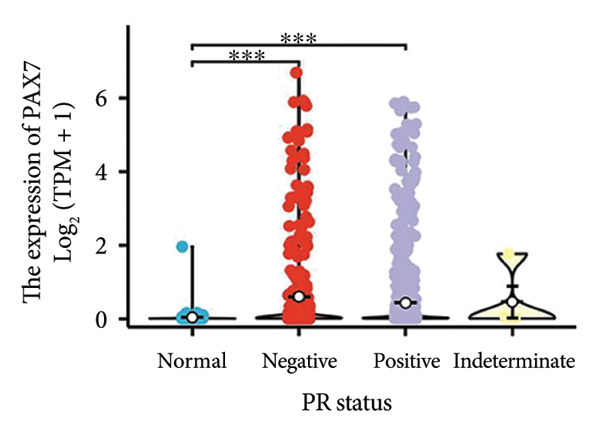
(g)

**Figure:**
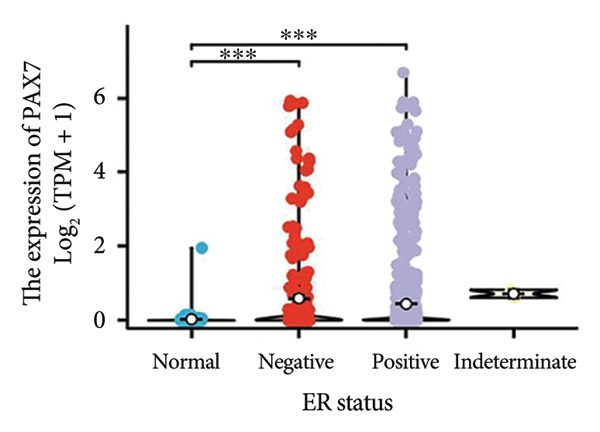
(h)

**Figure:**
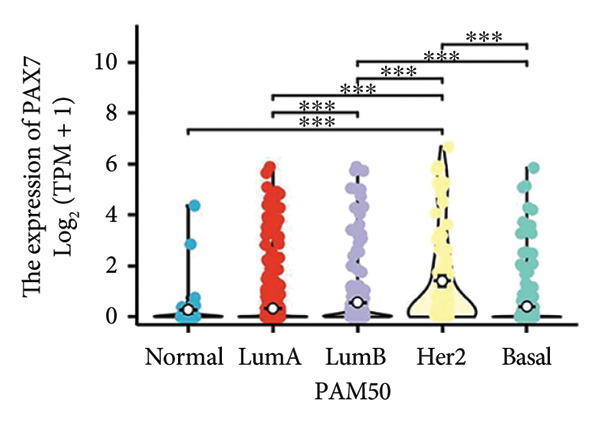
(i)

### 3.3. Relationship Between PAX7 Expression and Survival

To investigate the potential impact of PAX7 expression on patient survival, we utilized the GEPIA database to stratify breast cancer patients into groups exhibiting high and low PAX7 expression. Kaplan–Meier survival analyses indicated that the OS of patients in the high PAX7 expression cohort was markedly inferior to that of the low expression cohort (*p* < 0.01) (Figure 3(a)). Furthermore, Kaplan–Meier analysis leveraging the TCGA database was performed to elucidate the relationship between PAX7 expression and breast cancer prognosis. Figures 3(b) and 3(c) further examine the correlation between PAX7 expression and DSS as well as PFI, revealing that elevated PAX7 expression was associated with reduced DSS and PFI (*p* < 0.05). Prognostic subgroup evaluations depicted in Figures 3(d), 3(e), and 3(f) corroborated that high PAX7 expression is linked to unfavorable prognosis in breast cancer (*p* < 0.05).

Figure 3Prognostic relationship between PAX7 and breast cancer. (a) Examination of the association between PAX7 expression and OS in breast cancer patients via the GEPIA database. (b, c) Exploration of the connection between PAX7 expression and DSS as well as PFI in breast cancer patients using the TCGA database. (d–f) Prognostic subgroup evaluations of breast cancer patients with varying clinicopathological conditions.

**Figure:**
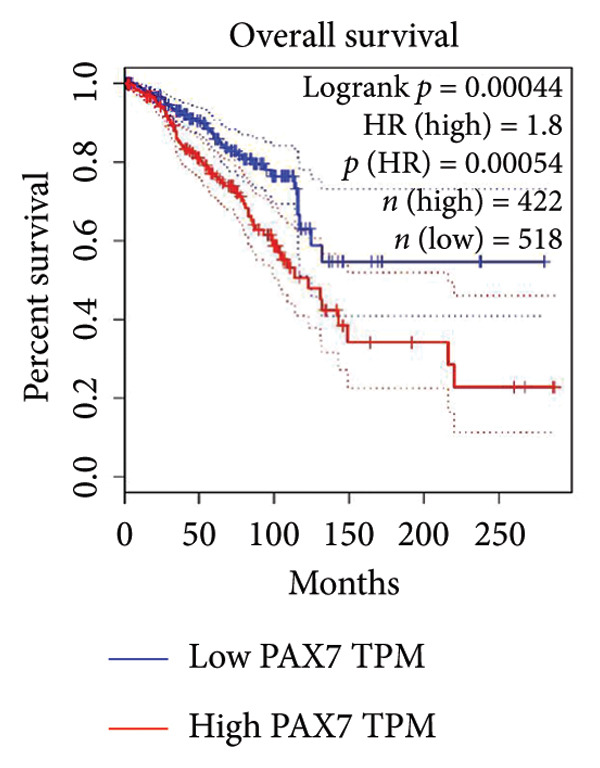
(a)

**Figure:**
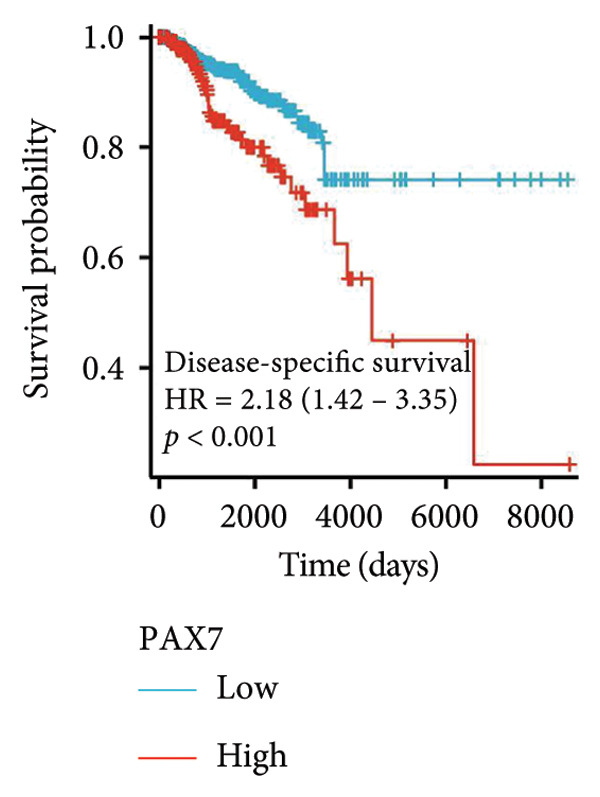
(b)

**Figure:**
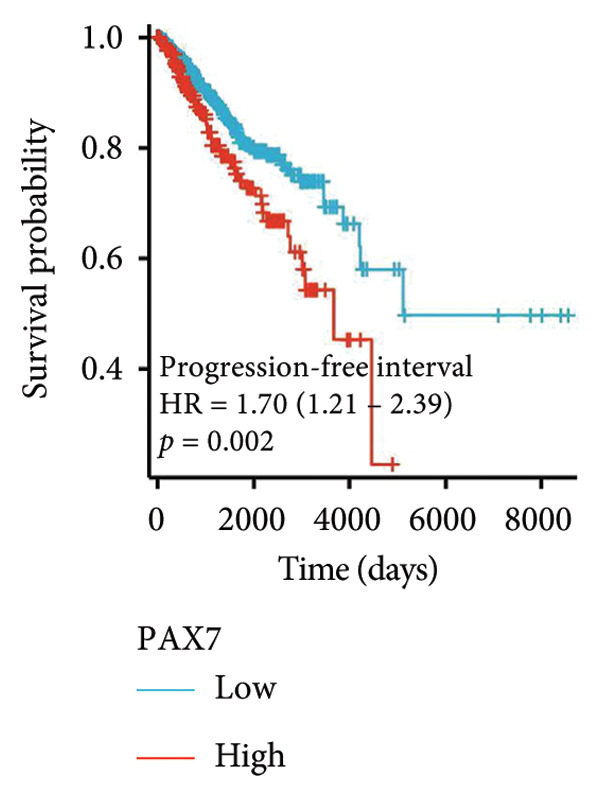
(c)

**Figure:**
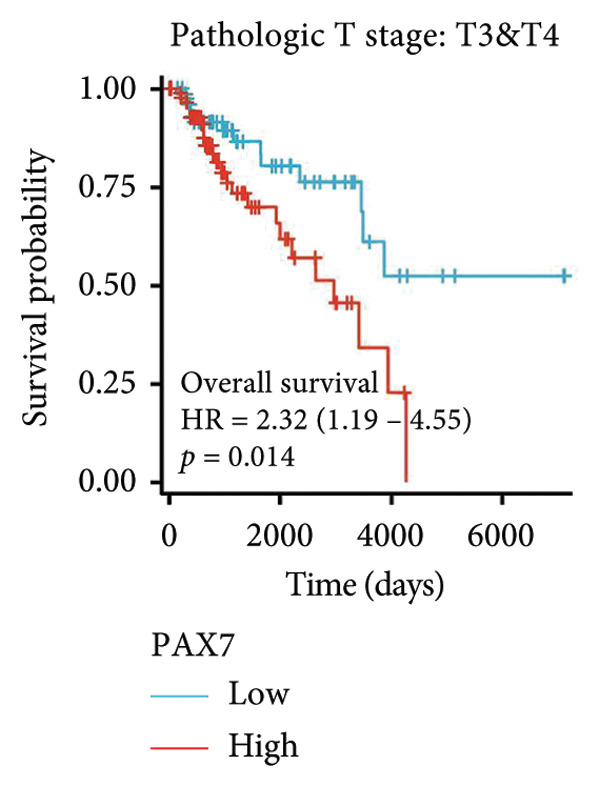
(d)

**Figure:**
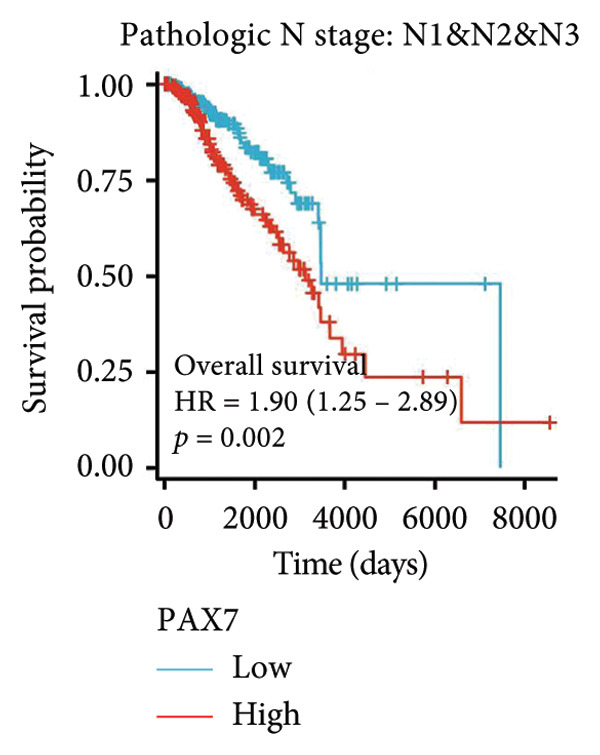
(e)

**Figure:**
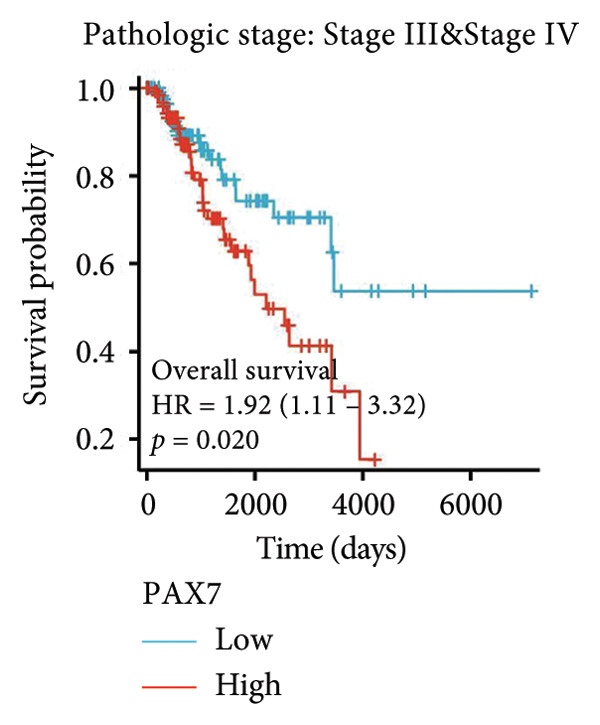
(f)

### 3.4. Prognostic Factor Cox Regression Analysis and Nomogram Construction

We evaluated the independent effects of PAX7 expression alongside other clinical characteristics on breast cancer prognosis employing the Cox proportional hazards model. PAX7 expression emerged as an independent prognostic factor for OS in breast cancer patients (HR = 1.459, 95% CI [1.027–2.072], *p* = 0.035). Additionally, age (HR = 2.157, 95% CI [1.511–3.079], *p* < 0.001), N staging (HR = 2.143, 95% CI [1.453–3.159], *p* < 0.001), and M staging (HR = 2.259, 95% CI [1.229–4.155], *p* = 0.009) were also identified as independent prognostic factors for breast cancer patients (Table 2). Figure 4(a) presents the nomogram designed for predicting OS, which integrates the independent prognostic factors identified in the multivariate Cox regression analysis to estimate the 1‐year, 5‐year, and 10‐year OS of breast cancer patients. The calibration curve illustrated in Figure 4(b) was utilized to validate the precision of these predictions.

**Table 2:** Univariate and multivariate Cox regression analyses of clinical characteristics associated with OS of breast cancer in TCGA.

Characteristics	Total (*N*)	Univariate analysis		Multivariate analysis	
		Hazard ratio (95% CI)	*p* value	Hazard ratio (95% CI)	*p* value
Age	1086				
≤ 60	603	Reference		Reference	
> 60	483	2.024 (1.468–2.790)	**< 0.001**	2.157 (1.511–3.079)	**< 0.001**
Pathologic T stage	1083				
T1&T2	908	Reference		Reference	
T3&T4	175	1.588 (1.096–2.301)	**0.014**	1.335 (0.882–2.022)	0.172
Pathologic N stage	1067				
N0	516	Reference		Reference	
N1&N2&N3	551	2.232 (1.563–3.189)	**< 0.001**	2.143 (1.453–3.159)	**< 0.001**
Pathologic M stage	925				
M0	905	Reference		Reference	
M1	20	4.266 (2.474–7.354)	**< 0.001**	2.259 (1.229–4.155)	**0.009**
PAX7	1086				
Low	542	Reference		Reference	
High	544	1.458 (1.053–2.017)	**0.023**	1.459 (1.027–2.072)	**0.035**

*Note:* The bolding was applied specifically to values where the *p*‐value was less than the predetermined significance level of 0.05.

Figure 4Construction of prognostic line graphs. (a) Nomogram for predicting OS at 1, 5, and 10 years. (b) Calibration curves for 1, 5, and 10 years.

**Figure:**
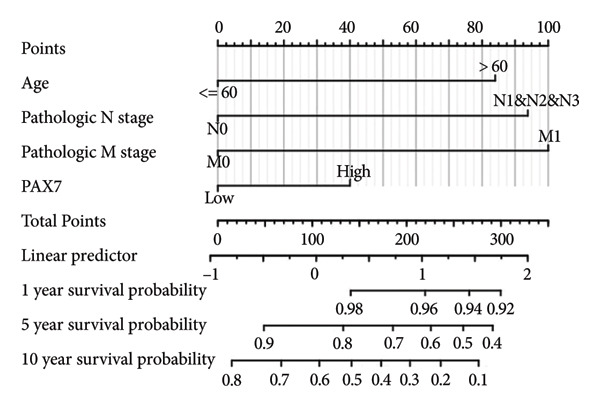
(a)

**Figure:**
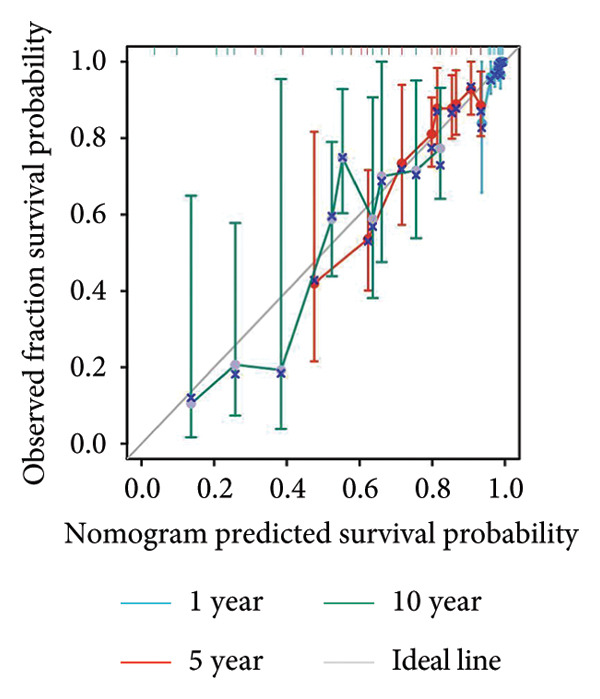
(b)

### 3.5. Functional Clustering Analysis of PAX7‐Related Genes

We executed a functional clustering analysis of genes coexpressed with PAX7. In BP (Figure 5(a)), processes closely related to cell division, such as organelle fission, nuclear division, and chromosome segregation, showed significant enrichment. This indicates that PAX7 is closely associated with the cell division process. In CC (Figure 5(b)), regions related to chromosomes, including condensed chromosomes, centromeric regions, and kinetochores, had prominent enrichment. This implies that PAX7 may play a crucial role in the formation and function of chromosome‐related structures. In MF (Figure 5(c)), multiple functions such as metal ion transmembrane transporter activity and tubulin binding were enriched. This suggests that PAX7 is involved in diverse molecular activities. In KEGG pathways (Figure 5(d)), pathways such as neurodegenerative diseases and the cell cycle were enriched, which points the way for subsequent research.

Figure 5Functional clustering of PAX7‐related genes. (a) Enrichment analysis of biological processes (BPs) of PAX7 coexpressed genes. (b) Enrichment analysis of cellular components (CCs) of PAX7 coexpressed genes. (c) Enrichment analysis of molecular functions (MFs) of PAX7 coexpressed genes. (d) Enrichment analysis of PAX7 coexpressed gene terms in the Kyoto Encyclopedia of Genes and Genomes (KEGG). ^∗∗∗^ *p* value < 0.001.

**Figure:**
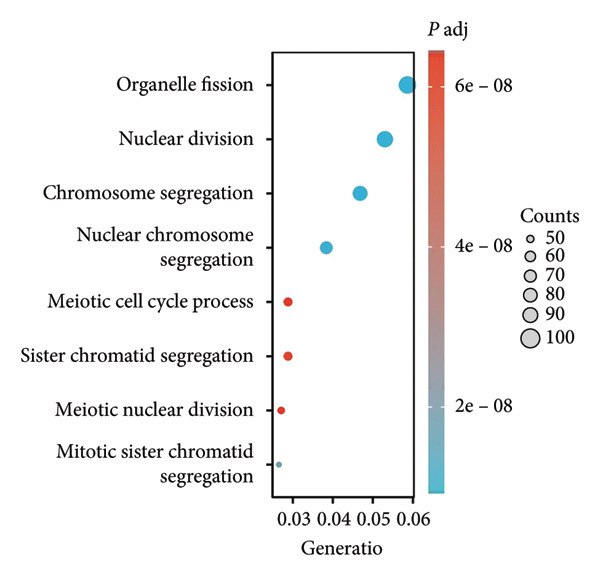
(a)

**Figure:**
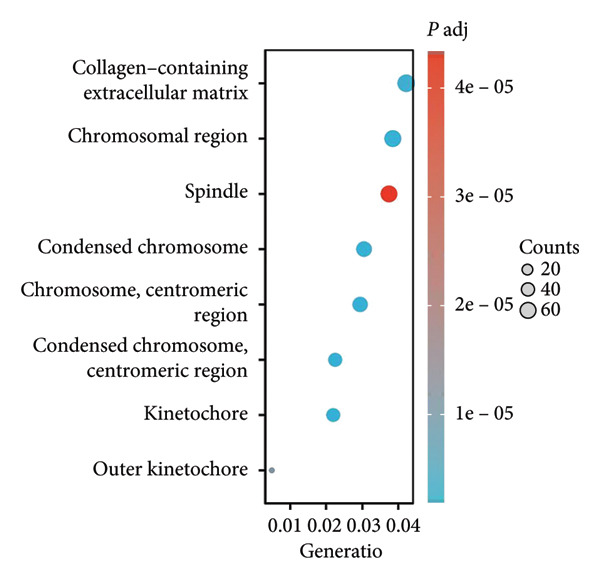
(b)

**Figure:**
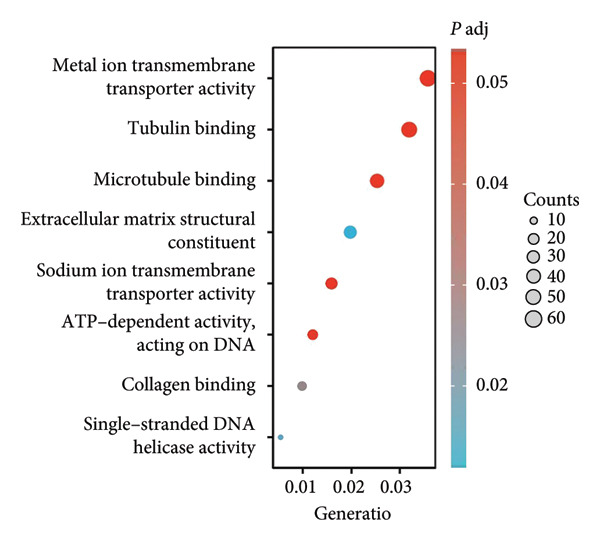
(c)

**Figure:**
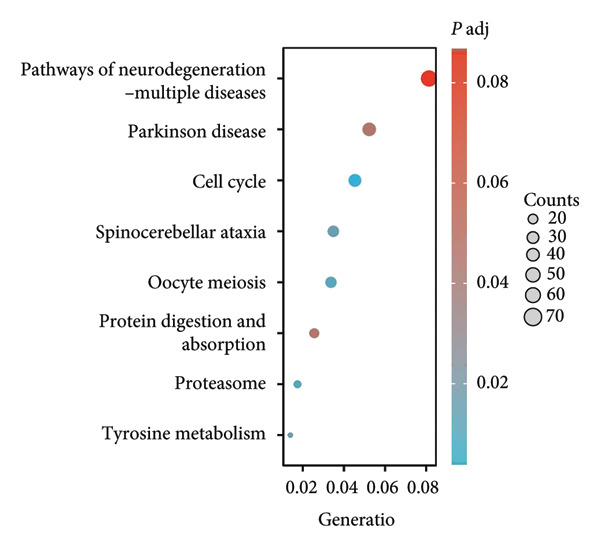
(d)

### 3.6. Relationship Between PAX7 Expression and Immune Infiltration

We examined the relationship between PAX7 expression and the extent of immune cell infiltration within the breast cancer microenvironment. Figure 6(a) demonstrates the correlation between PAX7 and immune cell infiltration. Figure 6(b) highlights the discrepancies in immune cell infiltration levels within the tumor microenvironment between patients exhibiting high and low PAX7 expression. Figures 6(c), 6(d), and 6(e) assess the infiltration levels of Th2 cells, TReg, and TFH in samples from patients with differing PAX7 expression levels, revealing a significant correlation between PAX7 expression and the infiltration levels of these immune cell types (*p* < 0.001). These findings suggest that PAX7 may exert an influence on breast cancer progression through its effect on immune cell infiltration.

Figure 6Relationship between PAX7 expression and immune infiltration in the breast cancer microenvironment. (a) The correlation between PAX7 and immune infiltrating cells in breast cancer. (b) Distinctive distribution of immune cells in patients with high PAX7 expression and low PAX7 expression. (c–e) Correlation between high and low PAX7 expression and the infiltration levels of Th2 cells, TReg, and TFH. ^∗^ *p* < 0.05, ^∗∗^ *p* < 0.01, and ^∗∗∗^ *p* < 0.001.

**Figure:**
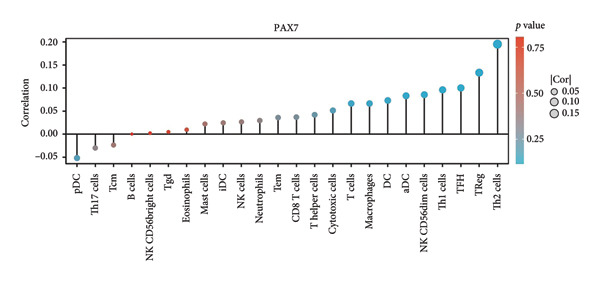
(a)

**Figure:**
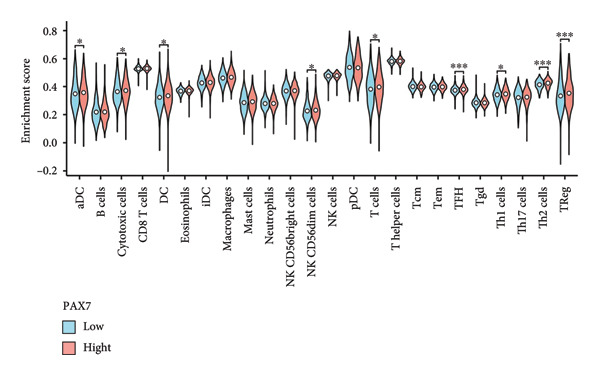
(b)

**Figure:**
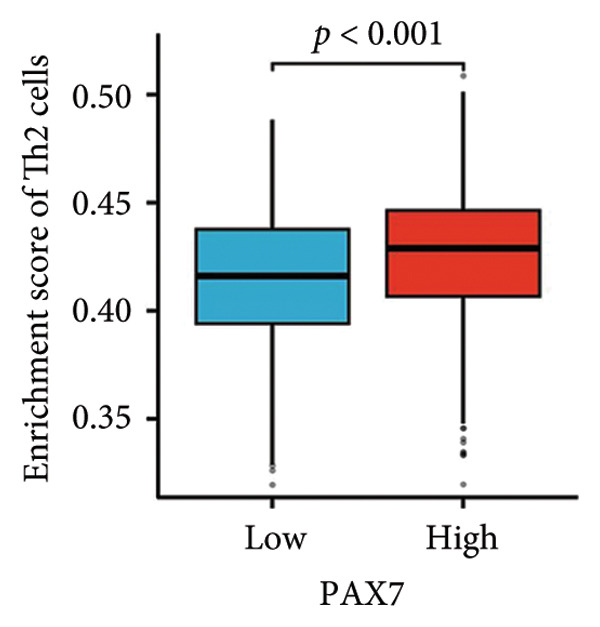
(c)

**Figure:**
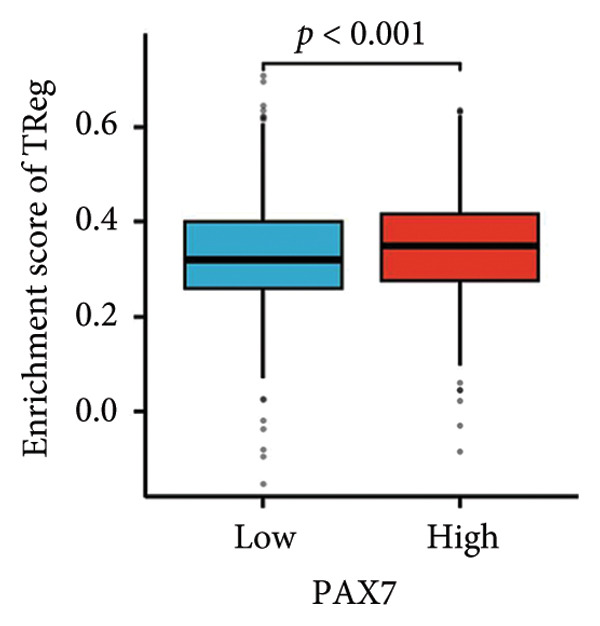
(d)

**Figure:**
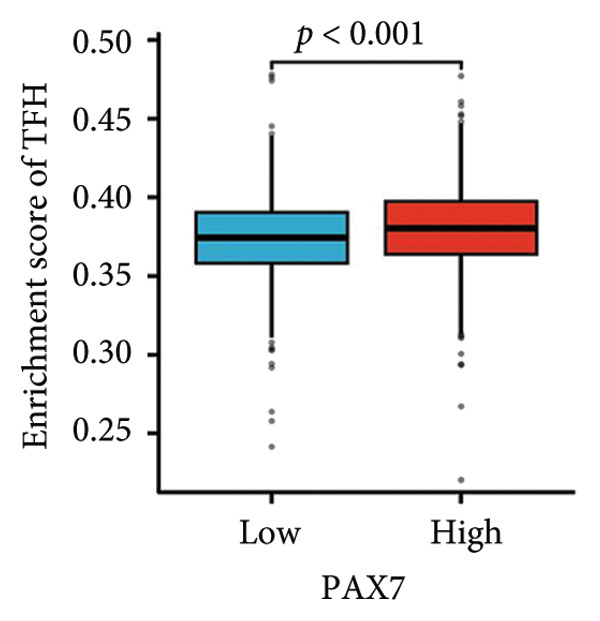
(e)

## 4. Discussion

Breast cancer represents one of the most prevalent malignant neoplasms among the female population, contributing to a considerable number of fatalities and economic strains annually [21]. As highlighted by global cancer statistics, the incidence of breast cancer continues to escalate, rendering it a critical concern within the realm of public health [22]. Despite the advancements in therapeutic interventions, including surgical procedures, radiotherapy, and chemotherapy, numerous patients continue to encounter significant hurdles such as drug resistance, recurrence, and metastasis [2, 23]. Consequently, the identification of novel biomarkers and therapeutic targets has great clinical importance in the improvement of patient outcomes and survival rates.

This study concerns breast cancer, with a particular emphasis on the implications of the PAX7 gene. To this end, we have performed our data analyses using public databases so that our data are full and reliable. In the present study, by applying sequential analysis procedures, it was determined that PAX7 expression in breast cancer tissues was remarkably higher than that in normal tissues; heightened expression showed a close relationship with poor prognosis, laying a very important foundation for the subsequent research work.

In our gene expression analysis, differential expression assessments were done using the limma package in R, and indeed, the results showed that PAX7 expression is significantly greater in breast cancer tissues when compared to their normal tissue counterparts. This disparity has indeed been pronounced, therefore underlining the potential of PAX7 as a biomarker in breast cancer. More importantly, the expression profile of PAX7 varies across different cancer types, further suggesting that its biological functions in breast cancer may be significantly specialized. Based on research into its molecular mechanisms, PAX7 may play a crucial role in the regulation of critical BPs involved in the proliferation, differentiation, and apoptosis of the breast cancer cells through precision regulation of the downstream gene cascades [24]. Functional enrichment analysis revealed that PAX7‐coexpressed genes are strongly associated with cell cycle progression, particularly the CDK4/6‐Cyclin D axis, which is a key driver of G1/S phase transition [25]. This suggests that PAX7 may promote tumor proliferation by dysregulating cell cycle checkpoints, providing a mechanistic link to its prognostic significance.

Coupled with other well‐researched biomarkers such as HER2, ER, and PR, PAX7 can significantly enhance the specificity and accuracy of diagnosis in breast cancer. Clinical expression levels of PAX7 will be able to provide an important reference for clinicians when formulating more accurate and personalized treatment options.

Prognostic analysis by Kaplan–Meier survival plot obviously presented that OS, DSS, and PFI in the high expression levels of PAX7 were remarkably poorer. Indeed, it implies that PAX7 acts not only as a promising biomarker but also serves as an important determinant for estimating prognosis in patients with breast cancer.

Furthermore, immune response analysis revealed that the tumor infiltration of Th2 cells, TReg, and TFH was significantly higher in the PAX7‐highly expressed patients. This observation may indicate that PAX7 expression modulates tumor development either directly in the tumor cells or indirectly by influencing the infiltration of immune cells. Notably, PAX7 may mediate immunosuppression by upregulating cytokines such as IL‐4 and IL‐10, which are known to drive Th2 polarization and TReg recruitment [26]. This paracrine mechanism could create a permissive microenvironment for tumor progression, linking PAX7 overexpression to immune evasion.​ Cox regression analysis showed that PAX7 expression was an independent predictor of OS in breast cancer patients (HR = 1.459, 95% CI [1.027–2.072], *p* = 0.035). Meanwhile, age and N and M staging were also independent prognostic factors. In clinical settings, healthcare providers may leverage this information to formulate more aggressive and personalized treatment strategies for patients exhibiting elevated PAX7 expression, particularly those who are older and present with lymph node or distant metastasis.

Functional clustering analysis demonstrated that genes coexpressed with PAX7 were significantly enriched in BPs, CCs, and MFs, encompassing vital processes such as cell proliferation and migration. PAX7, as an autonomous prognostic factor, may influence the biological behavior of breast cancer cells and subsequently exert a considerable effect on patient prognosis by modulating various BPs including cell cycle progression and apoptosis pathways [27, 28]. Intriguingly, comparative analysis with other PAX family members (e.g., PAX3 and PAX9) revealed that PAX7 exhibits unique oncogenic specificity in breast cancer. Unlike PAX3, which is linked to myogenic differentiation, or PAX9, which is associated with epithelial maintenance, PAX7’s enrichment in cell cycle pathways underscores its distinct role in driving breast carcinogenesis [29]. Future inquiries will necessitate the application of multidisciplinary approaches from cell biology, molecular biology, and related fields to investigate the specific molecular mechanisms by which PAX7 operates within these biological contexts, thereby providing a robust theoretical foundation for developing targeted therapeutic strategies.

This investigation also presents some limitations, such as dependence on publicly available databases, absence of laboratory validation, and the possibility of heterogeneity that may compromise the accuracy of the results. The variability within the datasets could lead to batch effects, thereby influencing the generalizability of the findings. Subsequent studies should focus on resolving these concerns, examining the biological functions of PAX7, and assessing its clinical relevance for the development of personalized treatment strategies in breast cancer.

## 5. Conclusion

This study systematically explored the expression and significance of the PAX7 gene in breast cancer by integrating public database resources and employing a series of bioinformatics analysis methodologies. The findings revealed that PAX7 expression in breast cancer tissues was markedly elevated compared to normal tissues. Survival analysis indicated that patients with high PAX7 expression experienced significantly shorter OS, DSS, and progression‐free survival. The Cox regression model identified PAX7 as an independent prognostic factor, while age, N stage, and M stage were also found to independently influence prognosis. Immune infiltration analysis revealed a significant correlation between PAX7 and the infiltration levels of Th2 cells, TReg, and TFH within the tumor microenvironment. Clustering analysis of PAX7 genes showed enrichment in cell division, chromosome regions, and pathways like the cell cycle. Subsequent investigations should thoroughly investigate the biological mechanisms underlying PAX7 while also enhancing its potential applications in the personalized treatment of breast cancer.

## Ethics Statement

The databases are publicly available, and our study was performed based on the guidelines of these databases. The research scheme complies with the requirements of scientific and ethical principles.

## Conflicts of Interest

The authors declare no conflicts of interest.

## Author Contributions

Xierzhati Aizezi: Conceptualized and designed the study, provided supervision, reviewed and finalized the manuscript, and acts as the corresponding author.

Bahatiguli Silafu: Contributed to data curation, formal analysis, and methodology; performed the bioinformatic analyses; and wrote the original draft.

Jinxing Huang: Conducted statistical computations, created visualizations and figures, and participated in writing the results and methodology sections.

First and second affiliations contributed equally to this work and are listed as co‐first affiliated institutions.

## Funding

No funding was received for this manuscript.

## Data Availability

The data supporting the findings of this study are publicly available from the following databases: The Cancer Genome Atlas (TCGA) (https://www.cancer.gov/tcga), The Human Protein Atlas (THPA) (https://www.proteinatlas.org/), the Gene Expression Profiling Interactive Analysis (GEPIA) platform (http://gepia.cancer-pku.cn/), and cBioPortal (https://www.cbioportal.org/).
